# The risk of distant metastases in patients with gynecologic cancers after surgery: a population-based study

**DOI:** 10.18632/aging.203773

**Published:** 2021-12-16

**Authors:** Yi-Hsuan Hsiao, Pei-Ni Chen, Min-Chien Hsin, Po-Hui Wang, Jing-Yang Huang, Shun-Fa Yang

**Affiliations:** 1 Department of Obstetrics and Gynecology, Changhua Christian Hospital, Changhua, Taiwan; 2 Women’s Health Research Laboratory, Changhua Christian Hospital, Changhua, Taiwan; 3 School of Medicine, Chung Shan Medical University, Taichung, Taiwan; 4 Institute of Medicine, Chung Shan Medical University, Taichung, Taiwan; 5 Department of Medical Research, Chung Shan Medical University Hospital, Taichung, Taiwan; 6 Department of Obstetrics and Gynecology, Chung Shan Medical University Hospital, Taichung, Taiwan

**Keywords:** gynecologic cancer, cervical cancer, uterus cancer, ovarian cancer, metastasis

## Abstract

The aim of the study was to determine the risk of distant metastases in patients with gynecologic cancers after surgery, including cervical, uterine and ovarian cancers. This is a retrospective study evaluating gynecologic cancer from 2009 to 2014 using population-based administrative datasets from the Health and Welfare Data Science Center (HWDC) and from The National Health Informatics Project (NHIP). A total of 1,464 gynecologic cancer patients, including 321 cervical cancer patients, 724 uterine cancer patients and 419 ovarian cancer patients, were analyzed retrospectively from 2009 to 2014. Among the cervical cancer patients, 173 (53.89%) received surgery only and 148 (46.11%) received surgery with radiotherapy /chemotherapy. Among the uterus cancer patients, 425(58.70%) received surgery only and 299 (41.3%) received surgery with radiotherapy /chemotherapy. Among the ovarian cancer patients, 81 (19.33%) received surgery only and 338 (80.67%) received surgery with radiotherapy/chemotherapy. Among patients with brain, liver or lung metastasis, cervical cancer patients have more cumulative metastasis-free survival than those ovarian cancer (p=0.0041). In analyzing liver metastasis based on primary cancer sites, cervical cancer patients and uterine cancer cases have more cumulative metastasis- free survival than those ovarian cancer (p<0.0001). In conclusion, ovarian cancer patients have higher risk of liver metastasis than cervical or uterine cancer. There were significantly different of pathological stage for cumulative metastasis-free survival among gynecologic cancer patients with brain or liver or lung metastasis. Pathological T stage remains the main predictive for distant metastasis of gynecologic cancer.

## INTRODUCTION

Gynecologic cancers are comprised of mostly ovarian cancer and malignancies of the cervix and uterus [1]. The recent literature about incidence of cervical cancer globally has been reported using data of cancer form 185 countries. This worldwide analysis for estimating the incidence of cervical cancer showed approximately 570,000 cases of cervical cancer in 2018; furthermore, the estimated age-standardized incidence was 13.1 per 100 000 women globally with ranging from less than 2 to 75 per 100,000 women among different countries. Cervical cancer was the fourth most common cancer in female worldwide. It was estimated that approximately 311, 000 deaths from the disease occurred in 2018 [2]. According to the study of cancer statistics for the year 2020, cervical, ovarian, and uterine cancer are the three most common types of gynecologic cancers that seriously threaten women’s life and health [3]. Ovarian cancer is the seventh most common cancer in women worldwide, the eighth most common cause of cancer death from the disease, with five-year survival rates below 45% [4]. Uterine cancer is increasing incidence and mortality [5, 6]. In less developed countries, uterine cancer is the 2nd most common gynecologic malignancy after cervical cancer [7]. Gynecologic malignant tumors, particularly cervical, uterine, and ovarian cancer have both high morbidity and mortality and threaten women’s life and health [8, 9].

Treatment of cervical cancer is based on the clinical stage. Early cervical cancers are treated in most cases with surgery. Chemoradiation and radiation treatment are for many locally advanced or metastatic cervical cancers. For metastatic and recurrent cervical cancer patients, a recent development in the field of immunotherapy, the response rates are still not >15% [10]. Treatment of uterine cancer includes surgery, radiation and chemotherapy. Most patients with endometrial cancer are diagnosed early. Hysterectomy is the treatment of choice in early stage of disease. Treatment for patients with advanced disease involves radiotherapy and/or chemotherapy [11]. Ovarian cancer is insidious in presentation, the most lethal gynecologic cancer and most women diagnosed at an advanced stage. Management of ovarian cancer includes a combination approach with surgery and chemotherapy [12]. Recurrence is usually incurable [13]. Therefore, there are strategies for treatment of these three kinds of gynecological cancers but questions remained for patients with distant metastasis. Particularly, it is important to determine those patients with gynecologic cancers after surgery developing distant metastases.

Uterus cancer can be divided into two main groups, including endometrial and mesenchymal malignancies [14]. Endometrial cancers are common and mesenchymal malignancies are rare with a poorer prognosis [14]. Cervical cancer has decreased due to vaccination, screening, early detection and treatment of cervical dysplasia or pre-invasive cancers [15]. Uterine cancer is the most common and ovarian cancer presents the highest mortality rate [15]. For the gynecologic cancers presenting different metastatic spread pattern, ovarian cancer disseminates throughout the peritoneum and upper abdomen; cervical and uterine cancers are found mostly in the primary organs [15]. Nevertheless, failure to control metastases result in a poor outcome [16]; distant metastasis is the major cause of cancer mortality [17]. Development of brain metastases is a real factor for overall cancer mortality in patients with advanced stage disease as prognosis remains poor in spite of multimodal treatments [18]. Liver metastases remain the challenge to successful management of malignant disease [19]. Lung metastases represent one of common sites of patients with advanced or recurrent endometrial cancer [20].

Gynecologic cancers may occur to women of all ages worldwide; the diseases often result in disruptions in physical and mental health, quality of life, and even early death [21, 22]. Some patients develop metastases after treatment even though with the strategies of standardized treatments of tumors in recent years [23].

The aim of the study was to determine the risk of distant metastases in patients with gynecologic cancers after surgery, including cervical cancer, uterus cancer and ovarian cancer.

## RESULTS

### Patients with gynecologic cancers

The cases were initially diagnosed cervical cancer (ICD-9:180, ICD-10:C53), uterus cancer (ICD-9:179, 182, ICD-10:C54, C55), ovarian cancer (ICD-9:183, ICD-10:C56). We identified the women diagnosed with cervical cancer (ICD-9:180 or ICD-10:C53), uterus cancer (ICD-9:179, 182, ICD-10:C54, C55), ovarian cancer (ICD-9:183, ICD-10:C56) from 2009 to 2014 in the cancer registry datasets. Initially, there were 2,058 cases with cervical cancer, 872 uterine cancer and 550 ovarian cancer. There were 2012 cases excluded, including 648 cases with pathological stage missing (study exclusion criteria including patients not undergoing surgery and these with limited external validity), 1276 cases with stage 0 (or *in situ* cancer), 8 cases without surgery, 84 death within one month. A total of 1464 patients were included in study groups, including 321 cervical cancer, 724 uterine cancer and 419 ovarian cancer.

### Outcome

Specific organ metastases were identified from gynecologic cancer cases. The gynecological cancer patients were diagnosed with lung (ICD-9: 197.0), liver (ICD-9: 197.7) or brain (ICD-9: 198.3) metastases. The characteristics among study groups were shown in Table 1. Most patients were diagnosed at age 40-59 years. Among the 321 patients with cervical cancer, 173 (53.89%) received surgery only and 148 (46.11%) received surgery with radiotherapy/chemotherapy. Among the 724 patients with uterus cancer, 425 (58.70%) received surgery only and 299 (41.3%) received surgery with radiotherapy /chemotherapy. Among the 419 patients with ovarian cancer, 81 (19.33%) received surgery only and 338 (80.67%) received surgery with radiotherapy /chemotherapy.

**Table 1:** Characteristics among study groups.

	**Cervical cancer N=321**	**Uterus cancer N= 724**	**Ovarian cancer N= 419**
Age			
<40	54 (16.82%)	54 (7.46%)	85 (20.29%)
40-59	199 (61.99%)	491 (67.82%)	231 (55.13%)
>=60	68 (21.18%)	179 (24.72%)	103 (24.58%)
Differentiation grade			
Well differentiated	38 (11.84%)	137 (18.92%)	19 (4.53%)
Intermediate differentiation	151 (47.04%)	250 (34.53%)	82 (19.57%)
Poorly differentiated	64 (19.94%)	143 (19.75%)	82 (19.57%)
Undifferentiated	4 (1.25%)	62 (8.56%)	107 (25.54%)
Unknown	64 (19.94%)	132 (18.23%)	129 (30.79%)
Pathological stage			
1, 2	264 (82.24%)	615 (84.94%)	244 (58.23%)
3	57 (17.76%)	109 (15.06%)	175 (41.77%)
Pathological T stage			
1, 2	316 (98.44%)	657 (90.75%)	254 (60.62%)
3, 4	5 (1.56%)	67 (9.25%)	165 (39.38%)
Pathological N stage			
0	267 (83.18%)	658 (90.88%)	337 (80.43%)
1, 2	54 (16.82%)	66 (9.12%)	82 (19.57%)
Co-morbidities			
Renal disease	3 (0.93%)	19 (2.62%)	17 (4.06%)
Hypertension	62 (19.31%)	232 (32.04%)	106 (25.30%)
Diabetics mellitus	29 (9.03%)	128 (17.68%)	48 (11.46%)
Hyperlipidemia	35 (10.9%)	144 (19.89%)	63 (15.04%)
Chronic hepatitis	15 (4.67%)	56 (7.73%)	34 (8.11%)
COPD	7 (2.18%)	25 (3.45%)	18 (4.3%)
Endometriosis	9 (2.8%)	96 (13.26%)	55 (13.13%)
Pelvic inflammatory disease	43 (13.4%)	98 (13.54%)	72 (17.18%)
Uterine myoma	56 (17.45%)	225 (31.08%)	93 (22.2%)
Adenomyosis	7 (2.18%)	75 (10.36%)	26 (6.21%)
Cancer treatment			
Surgery only	173 (53.89%)	425 (58.70%)	81 (19.33%)
Surgery with radiotherapy /chemotherapy	148 (46.11%)	299 (41.3%)	338 (80.67%)

**Figure 1:**
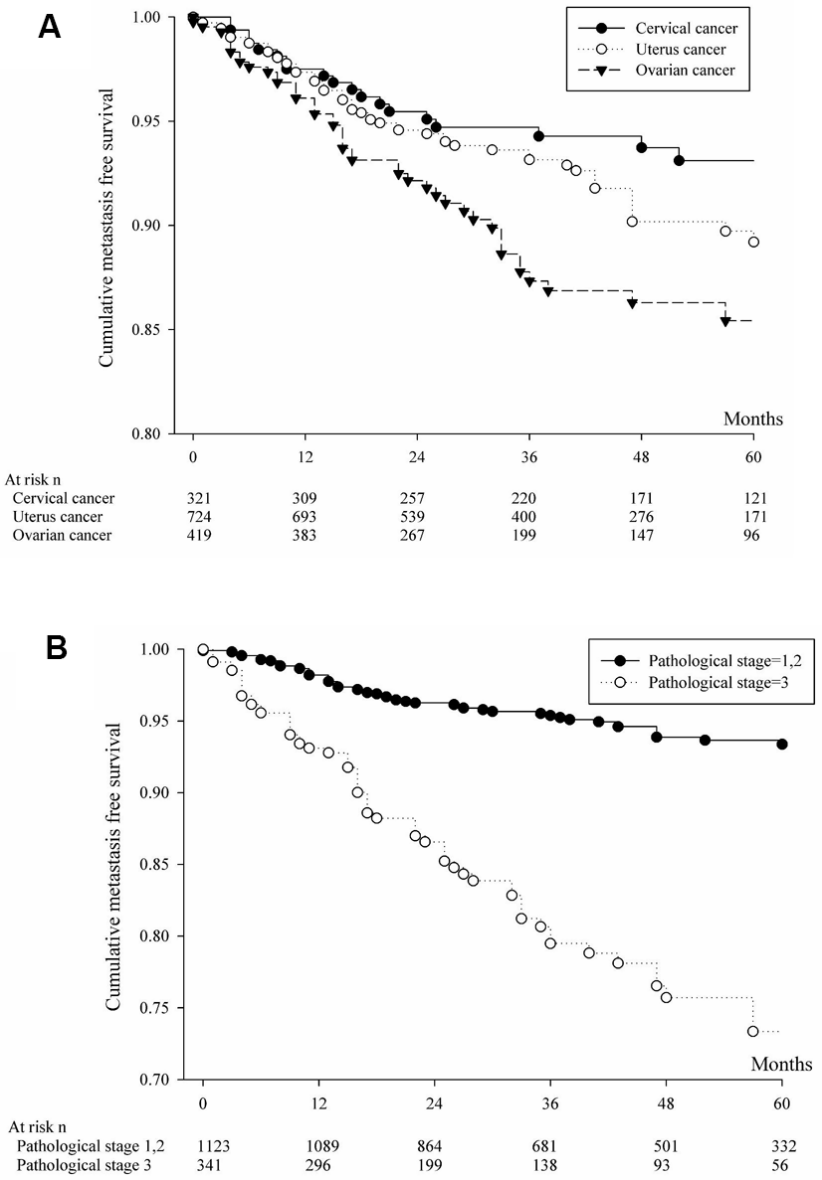
(**A**) Cumulative metastasis-free survival of patients with brain, liver or lung metastases from cervical, uterine or ovarian cancer. (**B**) Based on pathologic staging, cumulative metastasis-free survival was calculated for patients with brain, liver or lung metastases from cervical, uterine or ovarian cancer.

**Figure 2:**
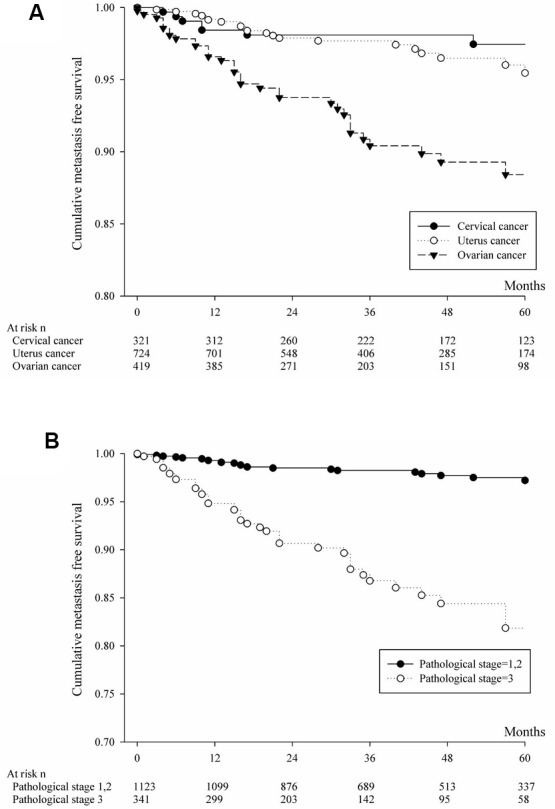
(**A**) Cumulative metastasis-free survival of patients with liver metastasis from cervical or uterine or ovarian cancer. (**B**) Based on pathologic staging, cumulative metastasis-free survival was calculated for patients with liver metastasis from cervical or uterine or ovarian cancer.

**Figure 3:**
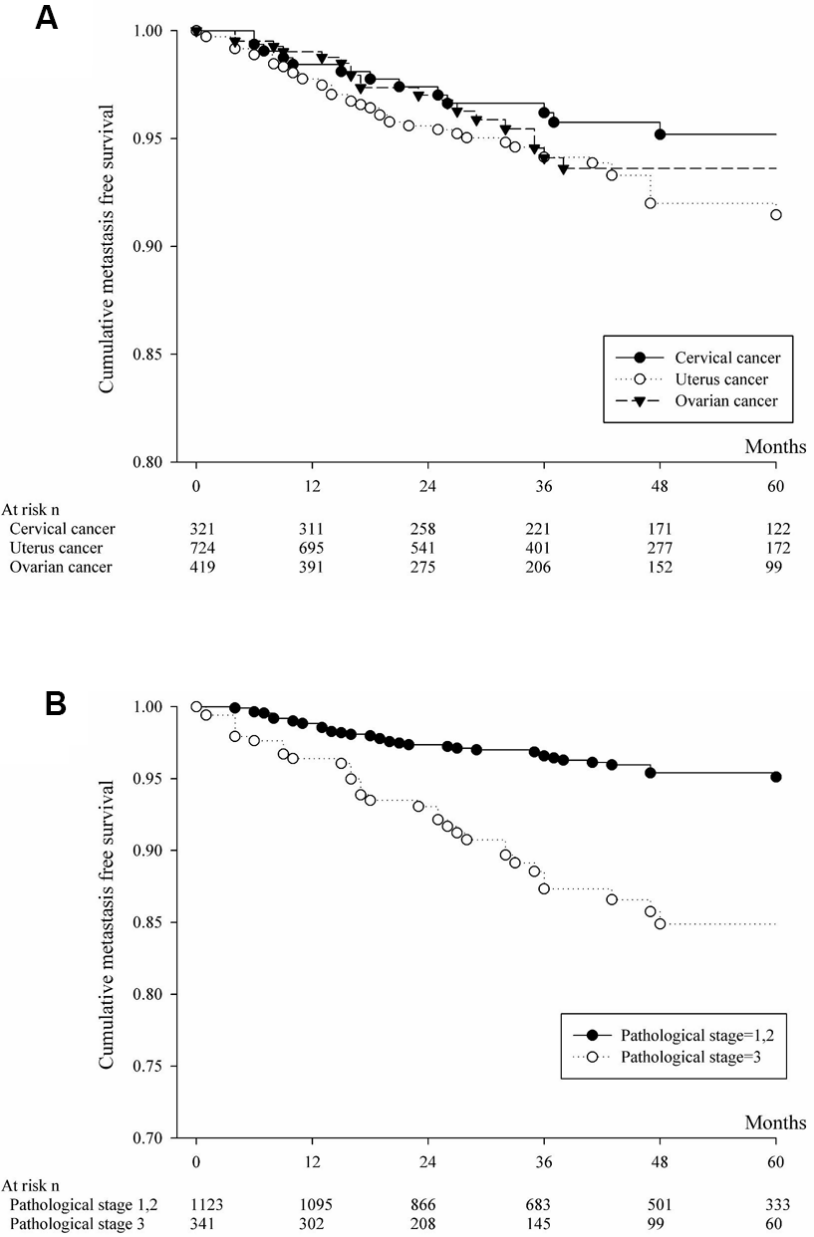
(**A**) Cumulative metastasis-free survival of patients with lung metastasis from cervical or uterine or ovarian cancer. (**B**) Based on pathologic staging, cumulative metastasis-free survival was calculated for patients with lung metastasis from cervical or uterine or ovarian cancer.

The analysis involving brain or liver or lung metastasis by primary cancer site was shown in Figure 1A and Table 2. Cumulative metastasis-free survival and multiple comparison by log rank test was shown by Table 2. Cervical cancer patients have more cumulative metastasis-free survival than those ovarian cancer (p=0.0041). Analyzing for brain or liver or lung metastasis based on pathological stage was shown in Figure 1B and Table 2. The patients with pathological stage 1or 2 significantly have more cumulative metastasis-free survival than those patients with pathological stage 3. The analysis involving liver metastasis by primary cancer sites was shown in Figure 2A and Table 3. Cervical cancer patients and uterine cancer cases have more cumulative metastasis-free survival than those ovarian cancer (p<0.0001). Analyzing for liver metastasis based on pathological stage was shown in Figure 2B and Table 3. The patients with pathological stage 1or 2 significantly have more cumulative metastasis-free survival than those patients with pathological stage 3 (p<0.0001). The analysis involving lung metastasis by cancer sites was shown in Figure 3A and Table 4. There was no significant difference in cumulative metastasis-free survival among the three cancer types. Analyzing for lung metastasis based on pathological stage was shown in Figure 3B and Table 4. The patients with pathological stage 1or 2 significantly have more cumulative metastasis-free survival than those patients with pathological stage 3 (p<0.0001). In analyzing brain metastasis, a total of 24 cases, there were 9 cervical cancer, 8 endometrial cancer and 7 ovarian cancer patients.

**Table 2:** Cumulative metastasis-free survival among gynecologic cancer patients with brain or liver or lung metastasis.

	**Cumulative metastasis-free survival**			
	**Cervical cancer**	**Uterus cancer**		**Ovarian cancer**
1 year	0.98 (0.95-0.99)	0.97 (0.96-0.98)		0.96 (0.94-0.98)
2 year	0.95 (0.92-0.97)	0.95 (0.93-0.96)		0.92 (0.89-0.94)
3 year	0.95 (0.91-0.97)	0.93 (0.91-0.95)		0.87 (0.83-0.91)
5 year	0.93 (0.89-0.96)	0.89 (0.86-0.92)		0.85 (0.81-0.89)
Multiple comparison by log rank test	Cervical vs. Uterus, p=0.8094			
	Cervical vs. Ovarian, p=0.0041			
	Uterus vs. Ovarian, p=0.1525			
	**Cumulative metastasis-free survival**			
	**Pathological stage=1,2**		**Pathological stage=3**	
1 year	0.98 (0.97-0.99)		0.93 (0.90-0.95)	
2 year	0.96 (0.95-0.97)		0.87 (0.82-0.90)	
3 year	0.95 (0.94-0.97)		0.79 (0.74-0.84)	
5 year	0.93 (0.91-0.95)		0.73 (0.66-0.79)	
log rank test	p<0.0001			

**Table 3:** Based on primary cancer site and pathologic staging, cumulative metastasis- free survival was analyzed for patients with liver metastasis.

	**Cumulative metastasis-free survival**			
	**Cervical cancer**	**Uterus cancer**		**Ovarian cancer**
1 year	0.98 (0.96-0.99)	0.99 (0.98-1.00)		0.97 (0.94-0.98)
2 year	0.98 (0.96-0.99)	0.98 (0.96-0.99)		0.94 (0.91-0.96)
3 year	0.98 (0.96-0.99)	0.98 (0.96-0.99)		0.90 (0.87-0.93)
5 year	0.97 (0.95-0.99)	0.95 (0.93-0.97)		0.88 (0.84-0.92)
Multiple comparison by log rank test	Cervical vs. Uterus, p=0.9972			
	Cervical vs. Ovarian, p<0.0001			
	Uterus vs. Ovarian, p<0.0001			
	**Cumulative metastasis-free survival**			
	**Pathological stage=1,2**		**Pathological stage=3**	
1 year	0.99 (0.99-1.00)		0.95 (0.92-0.97)	
2 year	0.99 (0.98-0.99)		0.91 (0.87-0.93)	
3 year	0.98 (0.97-0.99)		0.87 (0.82-0.90)	
5 year	0.97 (0.96-0.98)		0.82 (0.75-0.87)	
log rank test	p<0.0001			

**Table 4:** Based on primary cancer site and pathologic staging, cumulative metastasis- free survival was analyzed for patients with lung metastasis.

	**Cumulative metastasis-free survival**			
	**Cervical cancer**	**Uterus cancer**		**Ovarian cancer**
1 year	0.98 (0.96-0.99)	0.98 (0.96-0.99)		0.99 (0.97-1.00)
2 year	0.97 (0.95-0.99)	0.96 (0.94-0.97)		0.97 (0.95-0.98)
3 year	0.96 (0.93-0.98)	0.94 (0.92-0.96)		0.94 (0.91-0.96)
5 year	0.95 (0.92-0.97)	0.91 (0.88-0.94)		0.94 (0.9-0.96)
Multiple comparison by log rank test	Cervical vs. Uterus, p=0.2246			
	Cervical vs. Ovarian, p=0.8171			
	Uterus vs. Ovarian, p=0.6622			
	**Cumulative metastasis-free survival**			
	**Pathological stage=1,2**		**Pathological stage=3**	
1 year	0.99 (0.98-0.99)		0.96 (0.94-0.98)	
2 year	0.97 (0.96-0.98)		0.93 (0.90-0.95)	
3 year	0.97 (0.95-0.98)		0.87 (0.82-0.91)	
5 year	0.95 (0.93-0.96)		0.85 (0.79-0.89)	
log rank test	p<0.0001			

Table 5 showed the adjusted hazard ratio of distant metastases in study variables (brain or liver or lung metastasis). Among uterus and ovarian cancer patients, the cases with pathological T stage 3 or 4 have significant risk of distal metastasis than those patients with pathological T stage 1 or 2. The cervical and uterus cancer patients who received surgery with radiation/chemotherapy significantly have more distal metastasis than those patients with surgery only. A high adjusted hazard ratio (51.024 (6.328-411.441)) for renal disease of distant metastases in cervical cancer patients was shown in Table 5. This is concordant with the previous study. Hydronephrosis is possible associated co-morbidities (systemic diseases/complications) and with a trend to poor survival in patients with cervical cancer [24].

**Table 5:** The adjusted hazard ratio of distant metastases in study variables (brain or liver or lung metastasis).

	**aHR (95% C.I.) of distant metastases**		
	**In cervical cancer**	**In uterus cancer**	**In ovarian cancer**
Age			
<40	Ref	Ref	Ref
40-59	1.552 (0.151-15.983)	2.233 (0.551-9.040)	1.184 (0.501-2.798)
>=60	5.806 (0.579-58.186)	2.246 (0.502-10.044)	1.475 (0.489-4.448)
Differentiation grade			
Well differentiated	Ref	Ref	Ref
Intermediate differentiation	0.505 (0.053-4.776)	2.952 (0.926-9.414)	0.353 (0.080-1.551)
Poorly differentiated	2.430 (0.302-19.561)	2.122 (0.614-7.339)	0.841 (0.213-3.325)
Undifferentiated	-	7.344 (2.078-25.955)	0.389 (0.089-1.692)
Unknown	0.669 (0.054-8.221)	3.303 (0.926-11.786)	0.602 (0.150-2.422)
Pathological T stage			
1,2	Ref	Ref	Ref
3,4	4.560 (0.387-53.745)	2.996 (1.364-6.580)	4.11 (1.705-9.912)
Pathological N stage			
0	Ref	Ref	Ref
1,2	1.570 (0.453-5.442)	0.997 (0.445-2.234)	1.826 (0.911-3.66)
Cancer therapy			
Surgery only	Ref	Ref	Ref
Surgery+radiotherapy/chemotherapy	6.870 (1.243-37.972)	2.851 (1.293-6.287)	1.355 (0.494-3.715)
Co-morbidities			
Renal disease	51.024 (6.328-411.441)	0.887 (0.117-6.710)	-
Hypertension	0.947 (0.268-3.346)	1.058 (0.514-2.177)	0.440 (0.161-1.197)
Diabetics mellitus	0.659 (0.054-8.077)	0.758 (0.326-1.764)	1.781 (0.712-4.459)
Hyperlipidemia	0.448 (0.056-3.561)	0.930 (0.405-2.139)	0.768 (0.261-2.260)
Chronic hepatitis	-	0.849 (0.319-2.258)	1.170 (0.434-3.150)
COPD	-	2.861 (0.785-10.432)	0.954 (0.116-7.856)
Endometriosis	-	1.153 (0.190-6.994)	1.228 (0.344-4.389)
Pelvic inflammatory disease	0.686 (0.175-2.683)	1.859 (0.939-3.681)	1.143 (0.562-2.325)
Uterine myoma	2.540 (0.755-8.552)	0.697 (0.371-1.313)	1.830 (0.894-3.746)
Adenomyosis	8.483 (0.708-101.674)	1.254 (0.170-9.240)	1.063 (0.195-5.786)

## DISCUSSION

This study revealed the risk of distant metastases in patients with gynecologic cancers after surgery. Pathological stage remains the main predictive for distant metastasis of gynecologic cancer. Ovarian cancer patients have higher risk of liver metastasis than cervical or uterine cancer. There was no significant difference of risk of lung metastasis among the cervical, ovarian or uterine cancer. Among patients with brain, liver or lung metastasis, cervical cancer patients have higher cumulative metastasis-free survival than those ovarian cancer; the patients with pathological stage 1or 2 significantly have higher cumulative metastasis-free survival than those patients with pathological stage 3.

It was reported by a recent study that patients with cervical cancer, poorly differentiated carcinoma and early stage disease lead to brain metastases; among patients with endometrial cancer, high grade carcinoma and advanced stage disease are high risk for brain metastases [25]. In our study, most patients of uterine cancer developed distant metastases were those with undifferentiated tumor group; there were no significant differences in differentiation grade among the cervical cancer or ovarian cancer patients developing distant metastases. Comorbidity was common among patients with an advanced stage of cancer and played impact on mortality [18]. In our study, the most common co-morbidity was hypertension, 19.31% of cervical cancer, 32.04% of uterine cancer and 25.30% of ovarian cancer patients (Table 1).

In our study, patients with brain metastasis, a total of 24 cases, there were 9 cervical cancer, 8 endometrial cancer and 7 ovarian cancer patients. For reasonable statistic, the analysis of based on primary cancer site and pathologic staging, cumulative metastasis-free survival was not performed. Different survivals and prognostic factors varied in cervical cancer patients with different patterns of distant metastases [26]. The proportion of lung, liver, and brain metastases were identified in 59%, 16% and 2% of cervical cancer patients [27]. In the previous study, brain metastasis from cervical carcinoma is rare and the incidence is among cervical carcinoma patients of 0.6% [28]. The incidence of brain metastasis from cervical cancer amounts to mere 0.5 % although brain metastasis is the most common intracranial tumor [29]. The occurrence of brain metastasis has increased because overall survival in uterine cervical cancer was improved [30]. Brain metastases from ovarian cancer is a rare condition; advanced disease stage and high-grade serous ovarian cancer are common features among patients developing brain metastases [31].

In our study, higher cumulative metastasis-free survival for patients with liver metastasis in cervical cancer or uterine cancer than ovarian cancer patients; patients with stage 1,2 had higher cumulative metastasis-free survival than those with stage 3. Liver metastasis in cervical cancer are not common and present poor prognosis [32]. The investigators demonstrated that the site of metastasis is associated with overall survival and the patients with liver metastasis signifying particularly poor overall survival [33]. Liver metastasis was an independent prognostic factor for overall survival [33]. Median overall survival and median Progression-free survival for patients with liver metastasis were 6.8 and 3.7 months, respectively [33].

In our study, for patients with gynecologic cancer developing lung metastasis, there was no significant difference in cumulative metastasis-free survival among cervical cancer, uterine cancer or ovarian cancer; patients with stage 1,2 had higher cumulative metastasis-free survival than those with stage 3. The number of lung lesions at diagnosis was significantly associated with overall survival and event-free duration [34]. The weak relationship between the incidence of lung metastasis and the initial stage was reported by the recent study involving cervical cancer patients who developed lung metastasis after surgery, systemic chemotherapy or postoperative concurrent chemoradiation [34].

Ovarian cancer is one of the most common malignant tumor in women [35]. This disease is the most lethal gynecologic cancer; diagnosed at advanced stage among the majority of ovarian cancer patients [35]. The proportion of lung, liver and brain metastases were identified in 38%, 57% and 1% of ovarian cancer patients [27]. Lung metastasis is an independent risk factor affecting the prognosis of ovarian cancer patients [36].

Limitations of our study are as follows. For limited number of patients especially in advanced tumor stages, cumulative metastasis-free survival of patients with brain metastasis from cervical or uterine or ovarian cancer was spared. Clinical tumor stage (FIGO), radicality of resection of patient refusal, palliative resection, and treatment of distant metastases were not concerned. It was unknown whether patients completed neoadjuvant and/or adjuvant treatment. Distant metastases and survival were not linked.

In conclusion, the present study showed that ovarian cancer patients have higher risk of liver metastasis than cervical cancer or uterine cancer. There was no significant difference of risk of lung metastasis among the cervical, ovarian or uterine cancer. Pathological stage remains the main predictive for distant metastasis of gynecologic cancer.

## MATERIALS AND METHODS

### Data source

The National Health Informatics Project was established for promotion of sharing health-related data, protection of privacy, and reducing duplication in study work. The NHIP was regulated by the Health and Welfare Data Science Center in Taiwan (https://dep.mohw.gov.tw/DOS/np-2497-113.html). The NHIP provide the linkage of population-based administrative datasets, that including national health insurance database, cancer registry datasets and death registry datasets. This study was approved by the IRB of Chung Shan Medical University Hospital (the IRB approved number: CS17129).

### Patients with gynecologic cancers

Comprehensive information regarding clinical detailed and diagnostic codes of patients was based on the International Statistical Classification of Diseases and Related Health Problems codes ICD-9 (ninth revision, clinical modification) or ICD-10 (tenth revision, clinical modification). The inclusion criteria were as follows. The female patients with the first cancer diagnosed were cervical, uterine or ovarian cancer. The women diagnosed with cervical cancer (ICD-9:180 or ICD-10:C53), uterus cancer (ICD-9:179, 182, ICD-10:C54, C55), ovarian cancer (ICD-9:183, ICD-10:C56) from 2009 to 2014 in the cancer registry datasets were identified.

There are 2 staging systems for gynecologic cancers, including the tumor, node, metastasis (TNM) system and the International Federation of Obstetrics and Gynecology (FIGO) system [37]. The TNM system is produced by the International Union Against Cancer (UICC) (AJCC) and the American Joint Committee on Cancer. The FIGO revised the staging system for cervical cancer in 2018 [38]. Recently, the AJCC, in conjunction with the UICC, released an update of AJCC’s TNM staging for cervical cancer (version 9) [39]. Pathological stages can be determined from the findings at surgery, including the size of the tumor (T), the spread to nearby lymph nodes (N) and the metastasis to distant sites (M), namely Pathologic Tumor Category (pT), Pathologic Lymph Node (pN) Category and Pathologic Metastasis (pM) Category [37].

### Exclusion criteria

In the study, exclusion criteria are the cases of carcinoma *in situ*, any metastasis found within one month while the cases were diagnosed (not appropriately for analysis as metastasis occurred probably when diagnosed), or expired within one month while the cases were diagnosed cancer (not suitable for analysis because of too short interval follow-up). There were 1304 were excluded. The total number was of 754 cases included in our study.

### Definition of distant metastases

Distant metastasis, cancer spreading from the original (primary) tumor to distant organs or distant lymph nodes, was consented to be one of the significant characteristics in the advanced cancer. The site of distant metastases affected survival in metastatic cancer [36]. Little is known about the location patterns of distant metastatic and progresses in gynecologic cancers after surgery. The development of distant metastases at varied location and outcomes of ovarian, uterine, and cervical cancer patients after surgery were determined.

All the included cases were followed up one month later the diagnosis of cancer until the metastasis occurred. The metastasis included any metastasis (ICD-9: 196,197,198), liver metastasis (ICD-9: 197.7), brain metastasis (ICD-9: 198.3) and lung metastasis (ICD-9: 197.0) were investigated. As different gynecologic cancers or stages could exist different survey interval, it could lead to varied risk of metastasis.

### The explanatory variables

In this study, we collected the variables, including cancer sites, age, grade, pathological stage, pathological T, N stage, treatment of cancer, and co-morbidities, that might be associated with the distant metastases in these patients. Except co-morbidities, variables were from cancer registry database. The data, with great accuracy, were judged and recorded by pathologists and professional medical personnel. The co-morbidities were identified (by at least 2 outpatient visits or any admission record) from the national health insurance datasets, the accuracy of diagnosis with co-morbidity was varied between different diseases. However, the co-morbidities listed in this study had been published in previous Taiwan national health insurance research.

### Statistical analysis

Chi-square test was used to determine the homogenous of baseline characteristics among 3 gynecologic cancer groups. The incidence rate (95% confidence interval, C.I.) of any distant metastases was calculated by using the PROC GENMOD with Poisson regression and stratified by study groups, pathological stage, and cancer treatment. Furthermore, the incidence of liver, and lung cancer metastases was also counted. The sub-distribution hazard function was used to generate the cumulative probability of distant metastases since follow up. The competing Cox regression model was performed to estimate the adjusted hazard ratio, when considered the covariates of cancer sites, age, differentiation grade, pathological stage, pathological T, N stage, treatment of cancer, and co-morbidities. Statistical analyses were performed using SAS software version 9.4 and p<0.05 was considered statistically significant.
